# ‘You put up a certain attitude’: a 6‐year qualitative study of emotional socialisation

**DOI:** 10.1111/medu.13650

**Published:** 2018-07-30

**Authors:** Melissa Bolier, Karolina Doulougeri, Joy de Vries, Esther Helmich

**Affiliations:** ^1^ Academic Medical Center University of Amsterdam Amsterdam the Netherlands; ^2^ Department of Industrial Engineering and Innovation Sciences Section of Philosophy and Ethics Eindhoven University of Technology Eindhoven the Netherlands; ^3^ TBL Academie Abcoude the Netherlands; ^4^ Center for Education Development and Research in Health Professions University Medical Center Groningen Groningen the Netherlands

## Abstract

**Context:**

Emotions play a central role in the professional development of doctors; however, research into how students are socialised to deal with emotions throughout medical school is still lacking.

**Objectives:**

This study aimed to gain a better understanding of the emotional socialisation of medical students (e.g. how they learn to express and respond to emotions evoked in clinical practice in the process of becoming a doctor).

**Methods:**

In this longitudinal study, 12 medical students participated in annual, individual, semi‐structured interviews, capturing the full 6‐year medical school period. We carried out a thematic analysis, which was iterative and inductive.

**Results:**

The socialisation of emotion in the process of becoming a doctor happens in a complex interplay between student and context. We identified two modes of emotional socialisation (e.g. explicit and implicit teaching about emotions), the latter including how the people observed by students express their emotions and how they respond to the emotions expressed by students. Although the main message conveyed to students still seemed one about hiding or suppressing emotion, we found that students were able to identify and build upon the emotional expression and responses they observed in positive role models and managed to create their own opportunities to express their emotions. We found large differences between students in how they perceived, presented and developed themselves.

**Conclusions:**

Students differ in how they respond to and what they need from their environment and thus may benefit from tailored supervision in learning how to experience, express and respond to emotion. Providing students with real and authentic responsibility for patients and allowing them time and opportunity to talk about emotion might help them to create an emotional space.

## Abstract 2

The authors explore the socialization of emotions in the development of new doctors and show it to be an active process with a strong interdependence between individual and context.

## Introduction

Emotions are a fundamental part of every human being and influence social behaviour.^1^ As such, they are increasingly recognised as playing a central role in the professional development of doctors and other health care providers.^2^, ^3^, ^4^ Emotions direct how students experience and behave in certain situations, for instance when feeling excitement at a resuscitation or sadness when breaking bad news to a patient. Openly expressing those emotions may influence how others perceive you as a student, which might be a reason to deliberately manage these feelings.^5^ Within the sociocultural environment, in the interaction with both patients and colleagues, students learn about emotions and what is expected from them as medical students and future doctors.^5^ The problem is, however, that emotions do not get explicit attention in most medical curricula^6^, ^7^ and that we still fail to adequately support medical trainees in their emotional development.^8^ Although there are some published cross‐sectional accounts of emotions in medical students,^9^, ^10^, ^11^ and some quantitative studies suggesting a decline of empathy in medical school,^12^, ^13^, ^14^ qualitative longitudinal research exploring how students learn about emotion throughout medical school is still lacking, which is an important gap in the literature.^13^ If we knew more about how students are being socialised to experience and express emotions in clinical training in practice settings, we could develop more tailored approaches to support them.

In this study, we describe the longitudinal emotional development of medical students, as a follow‐up on our earlier cross‐sectional studies of the emotional experiences of medical students in early clinical placements.^2^, ^15^, ^16^ In those previous studies, we found that most experiences evoking emotion were related to patient care (e.g. witnessing patient distress or recovery and developing a professional relationship with patients).^15^ To a lesser extent, students mentioned emotions evoked by situations challenging their evolving professional identities and their positions as learners.^15^ We found major differences between students in how they gave meaning to their emotions and engaged in patient care, resulting in the description of four different ‘paradigms’ of lived experience: feeling insecure; complying; developing, and participating.^2^, ^17^ In their early clinical experiences, medical students found themselves facing several tensions evoking emotion: idealism versus reality; critical distance versus adaptation; involvement versus detachment; feeling versus displaying; finding versus not finding a role, and participation versus non‐participation.^2^, ^16^ We described how, most often, students were able to resolve those tensions in a positive way, but that sometimes their emotional struggles resulted in cynicism or detachment.^16^

This last finding reflects the early work of sociologists interested in the emotional socialisation of medical students, such as Hafferty,^18^ Smith and Kleinman,^19^ and Becker et al.,^20^ who showed how, at least in a major part of the past century, students in medical schools learned to hide their feelings and adopt strategies to manage their emotions about bodily contact (e.g. using clinical language and reducing the body to its anatomical parts), all leading to the development of objectifying and dehumanising attitudes towards their patients. More recently, MacLeod carried out a critical discourse analysis, explaining how medical students develop their professional identities by negotiating the competing discourses of competence and caring, in which competence tends to be associated with the suppression of emotions, most importantly uncertainty and anxiety.^21^ Based on a slightly different type of critical discourse analysis, building on figured worlds theory, Dornan et al.^22^ described the interplay between medical students’ emotions, professional identity development and power, advocating for the introduction of a ‘discourse of emotions’, which should help to make it legitimate to address emotions in medical training and clinical practice. In their social interactionist study of the sharing of emotions by medical students, de Vries‐Erich et al.^8^ found that students were reluctant to talk about emotions, in particular in formal training settings, and described how they adjusted the presentation of themselves, or their performances, according to what they believed was expected from them. This literature finds common ground in that it reveals how power in the social world of medicine leads to the hiding or suppressing of emotions.

In this study, following recent calls for a renewed interest in the topic of emotional socialisation during medical training,^23^ with an emphasis on qualitative research methodologies,^24^ we decided to follow a small group of students on their journey through medical school with the aim of exploring how they learn about emotion in the powerful field of medicine. Based on psychological and educational research on childhood socialisation of emotion,^25^, ^26^ we identify emotional socialisation as a process in which students learn from others about emotions in three different ways: (i) direct responses to the emotions of the student; (ii) discussion or explicit teaching about emotions by others in the social context, and (iii) expression of emotions by others in the social context. The aim of this longitudinal qualitative study was to gain a better understanding of the socialisation of emotion in the process of becoming a doctor.

## Methods

### Context

This study was carried out in one medical school in the eastern part of the Netherlands. Like all medical schools in the Netherlands, it has a 6‐year curriculum, with the first 3 years, leading to a Bachelor's degree, being mostly theoretical, and the last 3 years, resulting in a Master's degree, largely clinical. Students enter their first clinical placements (e.g. a 4‐week attachment to nurses) in Year 1. During this placement, they work in hospitals or nursing homes as assistant nurses and provide basic patient care. The main goals are learning practical and communication skills, empathising with patients and developing professional behaviour. Students are enrolled in clinical skills training by the end of Year 3 to prepare them for entering clinical practice again at the beginning of Year 4, when they start their clerkships.

### Participants and data collection

In this longitudinal qualitative study, our main focus was on the emotions of medical students evoked by clinical experiences. For this purpose, the students were interviewed about their experiences annually, except for Year 2 because of the lack of clinical experience in that year. All interviews were conducted by the same trained qualitative researcher (EH), who actively maintained contact with the students over the years. The interviews all focused on the same topics: emotional experiences; responses to those situations of the student and others in the environment, and students’ personal and professional development throughout the years. Recurrent questions were as follows: Can you describe one of your most emotional experiences in clinical practice? What did you think/feel/do? How did other people respond? How did this impact your development as a doctor? The interviewer actively recalled what was discussed in previous interviews to explore how students had developed over time.

At the end of a 6‐year period, we had collected up to five in‐depth interviews for every student, each lasting approximately 1 hour. These interviews captured the range of clinical practice settings (i.e. from entering medical school until the final clerkship year). For the initial study, we purposively sampled 17 medical students (Year 1, 2010),^2^ of whom 12 participated in the second interview (Year 3, 2012), 11 participated in the third interview (Year 4, 2013) and eight stayed involved during clerkships until graduation (Years 5 and 6, 2014–2016). Between Years 1 and 4, two students left medical school and three others did not respond to further invitations. During follow‐up, one other student left medical school and three others stopped responding. We think the final sample, although small, offers diverse perspectives and student characteristics, such as differences in age, gender and previous and current experiences.

### Data analysis

All interviews were audiorecorded and transcribed verbatim by the first author (MB), who gave participants pseudonyms to maintain confidentiality. We used both transcripts and recordings during analysis, the latter being accessible only to the first and senior authors (MB and EH). To meet the goals of the study, we focused on what students experienced as emotional and how they dealt with this over the years. To analyse the large amount of data, we first selected those parts of the interviews that referred to emotions, emotional experiences or emotional development in any possible way. This was the first step of the thematic analysis we used to explore the data.

Thematic analysis is a commonly used qualitative method for ‘identifying, analysing and reporting patterns (themes) within data’.^27^ Although there are various styles of thematic analysis, as it can be used for various kinds of data, there is a basic system following three steps: descriptive coding; interpretive coding, and identifying overarching themes.^28^ We followed an inductive approach, where the themes identified were data‐driven without using a pre‐existing frame.

After having selected the relevant material, a medical student, as the lead researcher (MB), defined descriptive codes, returning to the full interviews several times to ensure there were no relevant parts missed, and then further refined her coding. As a next step, she clustered the descriptive codes, trying to give meaning to those clusters, which resulted in a set of interpretive codes. This was followed by the identification of key or overarching themes for the data as a whole. Each step was followed by discussion with the senior author (EH), who is an elderly care physician and medical educator. Codes and themes were refined together with the senior author in a collaborative analysis and then presented and discussed in the larger research group, which included a psychologist (KD) and an educationalist (JdV). Finally, the themes were put together in a big scheme including a time frame, allowing the researchers to study the longitudinal emotional development of the students in a systematic way.

### Translation strategy

Data were collected in Dutch but are presented in this paper in English. Moreover, one member of our research group (KD) does not speak or understand Dutch. Therefore, MB and EH needed to translate relevant parts of the interviews into English, which was followed by a discussion with the two other authors (KD and JdV) in order to reach a shared understanding of central ideas, codes and themes.^29^

### Ethics

At the start of this longitudinal study, ethical approval for education research was not yet required in the Netherlands, and therefore this project was not taken under consideration by the institutional review board. However, the management board of the medical school where the interviews took place granted permission and the students all gave written consent prior to the interviews. Participation was voluntary and kept confidential.

As the interviews were about emotional experiences, we were aware that it could be difficult or confrontational for the students. Moreover, participating in this kind of study may increase the awareness of the students, changing personal development and future experiences. Therefore, we explicitly offered support afterwards and the interviewer clearly indicated that she, or some other support person, was always available to talk again.

## Results

We followed 12 students over the years, some longer than others, exploring their emotional socialisation throughout medical school (Table 1). Students’ narratives included descriptions of intense emotional experiences, mostly related to confrontations with human fragility, but in later years increasingly including emotions related to their positions as learners and the development of their professional identities. In the interviews, students narrated how they learned about emotions from others in the social environment. We identified two modes of emotional socialisation, explicit and implicit teaching about emotions, the latter including how the people observed by students expressed their emotions and how they responded to the emotions expressed by students. Longitudinal accounts of four ‘exemplar’ students are presented to demonstrate how the interaction between student and context unfolds over time.

**Table 1:** Overview of demographics and data collected from the interviewed students

Name (pseudonym)	Gender	Age, years (at first interview)	Bachelor (Years 1–3)			Master (Years 4–6)		
			Year 1 (2010)	Year 3 (2012)	Delayed (2013)	Year 4 (2013)	Year 5 (2014/2015)	Year 6 (2016)
Bernhard	M	20	X	X		X	X	X
Gerard	M	18	X	X	X			
Inge	V	18	X	X		X	X	X
Lineke	V	19	X	X		X	X	
Marianne	V	19	X	X				
Saar	V	18	X	X		X	X	X
Sanne	V	20	X	X		X		
Sanneke	V	18	X	X		X	X	
Suzanne	V	21	X	X		X	X	X
Vera	V	18	X	X		X	X	X
Veronique	V	19	X	X		X	X	X
Wout	M	26	X	X	X			

In 2013, Gerard and Wout were still on the Bachelor's course. In 2014/2015, Gerard did not respond and Wout was still on the Bachelor's course.

### Explicit and implicit teaching about emotion

Teaching students how to deal with emotions in order to become doctors started in the first year of medical school, before they entered their first clinical placements, when students were taught about the expected behaviours between a doctor and patient: ‘You can have them [emotions], but you cannot show them. We are really working on that in our first year’ (Sanneke, Year 1). This explicit teaching of professional behaviour or professional distance continued in the third year, when specific messages were conveyed about ‘professional attitude’, implying that ‘you can't let them [patients] come too close’ (Saar, Year 3).

Throughout their clinical placements, students’ learning about expected professional behaviour became more implicit and largely occurred by observing the expression of emotion by other health care professionals during their work. One of the students described how he observed:

…how people respond to each other […] and I saw that when patients start to cry, that the nurses sometimes tend to look away, that they do not dare to accept that patients cry. (Wout, Year 1)

Overall, the main lesson conveyed to students seemed to be that they should learn to control or, more often, even suppress emotions. From observations in practice, students learned that a doctor should not be crying while breaking bad news: ‘so that is just a matter of learning to control that’ (Sanneke, Year 5). A student witnessed a resuscitation of a newborn where she found the doctors acting upon routine, not showing emotions ‘and I think that is a sign that you should put your emotions aside for a moment’ (Vera, Year 5). In the face of this dominant notion, students felt they should learn to put on a professional ‘face’ to hide their feelings, having many intense emotions, but hoping ‘that at least I do not show it’ (Inge, Year 4).

Students explicitly talked about how nurse preceptors sometimes were very supportive in their responses to the emotions that were evoked by being in clinical practice. In Year 1, students had their first experiences with washing patients and touching naked bodies. Initially, some of them mentioned fear and disgust:

I really didn't know I was afraid of this, but I found myself being […] scared to death; and the first two days, I really thought that, I very strongly felt I didn't want to touch them. (Sanneke, Year 1)

Students mentioned how nurse preceptors were comforting students, assuring them they were not unique in having those feelings, inviting them to sit down and take a pause or explicitly asking for emotions. For instance, after a student had witnessed the death of a patient, ‘when we left the room, my supervisor asked: and, how do you feel?’ (Inge, Year 1). Also in the clerkships, students explicitly mentioned emotional support from nurses. One interviewee reported that at a resuscitation:

…they invited [the patient's] children to enter the room, which made me feel very sad […], it was the first time for me to see this, I felt fully overwhelmed […], one of the nurses came to me and spent some time with me. (Sanneke, Year 6)

Students also reported instances in which registrars or residents showed helpful responses:

I was in the room with the family, when the resident was going to break the bad news that [the patient] would not recover, that she was going to die; and then you saw the husband breaking down, and then I … well, I don't know, if I, if I was in tears, there, in the room, but the resident mentioned afterwards that I seemed very touched, and I agreed, and he also seemed to have a hard time during the conversation, these are the things that you will remember […] it was nice, that he addressed it. (Veronique, Year 4)

Despite highly valuing the emotional support provided by nurses and medical trainees, however, students still tended to identify with what they had been explicitly taught before: that doctors should not openly express their emotions. Although, during a resuscitation, the student was ‘really glad to see that several doctors had tears in their eyes’, her main observation was that ‘they were able to suppress it better than me of course, seeing it for the first time’ (Sanneke, Year 6).

Students seemed to be caught in a dilemma. They seemed reluctant to lose too much of their emotions, observing that ‘people who work here longer – I get the feeling their empathy decreases a bit; I just do not want that to happen for me’ (Suzanne, Year 1). They realised, however, that ‘when you do it daily it becomes some kind of routine’ (Suzanne, Year 1). Early in medical school, students emphasised that, despite getting used to certain emotions, they were not ‘walking through the hallways as a complete emotionless monster’ (Sanneke, Year 1). As a result of the explicit and implicit teaching about emotion, however, the language students used to talk about emotion increasingly started to reflect professional norms around emotion in the process of developing their professional identities:

I really have the feeling that I can keep more distance […] when you experience things more often, you put up a certain attitude or, yes not become immune but […] that's part of my profession. (Suzanne, Year 4)

### Emotional socialisation: the interplay between student and context

Students differed in how they described themselves, how they narrated their emotions and in how they responded to the explicit or implicit teaching about emotion. The interaction between student and context resulted in different processes of emotional socialisation over time. We will illustrate this by presenting the stories of four different students, who serve as ‘exemplars’, and as such also represent other participants.

Some students, with Lineke as an example, started medical school feeling very uncertain and overwhelmed by finding themselves in a new and unfamiliar situation. These students described as their most dominant emotions their uncertainties and fears about talking to patients and doing things on their own:

In the beginning, I found it very difficult, I am not someone who easily talks with people, that I just dare to do things, so I found it very hard. (Lineke, Year 1)

Feeling ‘very uncertain, afraid of doing things wrong’, Lineke explicitly needed support from others:

There really needs to be someone present saying, yes, you're doing it right, and, well, then I will do it, but doing something new on my own, I don't dare do that. (Lineke, Year 1)

For these students, it seemed important that their uncertainties were recognised and that responses from the people around them were supportive and reassuring, to help them overcome their anxieties.

After a few years, Lineke described how:

…in the beginning of the first clerkship […] I was really uncertain and did not dare to ask anything … [but] discovered that if I don't do it for myself, but for someone else, being the patient, I do much more […] I dare more. (Lineke, Year 5)

Despite still feeling uncertain about herself, she found that when identifying with the professional identity of a future doctor, feeling responsible for patients, she was able to overcome her fears. Unfortunately, when she was concerned about a patient looking very ill and insisted that something needed to be done, the doctors did not listen and the patient died the day after:

I was like gosh, now I did open my mouth and I've said something about it, several times […] and they've done nothing with it. I thought that was eh, I thought that was frustrating […] I thought it was really frustrating, because it was really one of the first times I said something about it that often. (Lineke, Year 5)

This response to Lineke, not listening when she finally dared to speak up, led to frustration and may lead students to back off again, finding themselves left with their initial uncertainties.

Other students, exemplified here by Bernard, were far less dependent on the context, stating in the beginning: ‘I'm not like that; I often think of something as emotional’ (Bernard, Year 1). After a few years of training, explicitly discarding external influences, Bernard announced: ‘I think that I have seen enough, that not much will change anymore in how I perceive myself as a doctor’ (Bernard, Year 4). Despite explicit communication skills training, ‘that did not add much, I always disliked it’, he explicitly stated: ‘I will not be sitting with a patient for minutes, that does not fit who I am as a person’ (Bernard, Year 6). Bernard described himself as someone who can ‘easily distance myself from the sadness of patients’ (Bernard, Year 4), not being ‘really blown away by the fact that patients die’ (Bernard, Year 6).

The only explicit emotions mentioned by Bernard were related to his responsibility for patients, which started right from the beginning, in his first clinical placement as a nurse assistant on an orthopaedic ward:

My biggest fear is that when I do something that someone's hip will dislocate […] that's my own biggest fear. […] Not that I'm constantly thinking about it, but I think when that happens, I will feel very bad. (Bernard, Year 1)

In his clerkships, emotions again were evoked by feelings of professional responsibility. Bernard became ‘honestly angry’ upon observing a physician breaking bad news ‘in the middle of the corridor, without any preparation’ (Bernard, Year 5). Feeling the need to take care of the patient, Bernard ‘just called the orthopaedic surgeon to ask for clarification’ and told him that ‘it would be correct […] to have a proper conversation with the patient and his family’ (Bernard, Year 5). Bernard expressed his anger regardless of the responses from others:

I think that also if someone might have tried to make the mistake of telling me that I was just a medical student, this would have turned out badly for him, I would have learned him a lesson. (Bernard, Year 5)

For both Lineke and Bernard, taking responsibility for patients apparently reflected one of the core values of being a doctor and thus formed an important reason to act upon emotion:

I feel this responsibility […] that as a doctor, you need to stand up […] and when you think a patient deserves a proper explanation, you may demand that from others. (Bernard, Year 5)

The interplay between student and context was fairly different for students who explicitly developed their professional identities in interaction with their environment, as exemplified here by Saar. Saar perceived and presented herself as much more emotional, for instance when feeling ‘really shocked’ upon her first interactions with patients in the nursing home, feeling sad about ‘that appearance and how they, the state they are in’ (Saar, Year 1). She felt attracted to the domain of palliative care but realised:

…the person is going to die, so if you let this come too close, you may fall apart yourself […] this likely will be a quest, because I always take things to heart. (Saar, Year 3)

Just like Lineke, Saar actively looked for emotional support from other people on the wards. In the nursing home, she reported:

[I] once felt a bit scared; this was with a man who was a, um, making sexual advances, he once came and stood quite close to me and I thought: ‘This doesn't feel good […] what is he going to do?’ (Saar, Year 1)

She shared her worries with the nurse preceptor, who taught her that inappropriate or threatening behaviours are ‘part of their disease, they don't deliberately act that way’ (Saar, Year 1). A few years later, when being confronted with a patient who got angry, Saar turned to her supervisor again, as she had done years before, and:

…talked with the family doctor about this interaction, and about the context, and why [the patient] reacted the way she did, and this helped me to understand. (Saar, Year 5)

Thus, Saar asked others to explicitly teach her about emotion. Based on her observations of the actual behaviours of others, and how they did or did not express emotions, however, she doubted whether she would fit into the culture of medicine:

…the atmosphere is really important […] if you are uncertain about something, that you should be allowed to share that, and that someone should be willing to help you instead of blaming you for what you don't know. (Saar, Year 5)

The influence of the clinical environment, indeed, was not always positive, for instance when Saar observed a trainee disclosing a medical error, which was discarded by the supervisor. This made her reflect on the importance of role‐modelling, perceiving this behaviour as:

…very bad; reflection is very important to prevent such errors, and when someone just laughs, this will lead to a feeling of insecurity […] now she learned that she should not care, that she should just go on and only laugh about it. (Saar, Year 6)

Saar was strongly aware of a possible tension between herself being emotional and sensitive to the emotions and behaviours of others, and different expectations from the clinical environment, for instance ‘at the ophthalmology department […] they are seeing a next patient every 5 minutes’ (Saar, Year 4). Saar eventually found a way to be true to herself by creating a specific role for herself as a clinical clerk:

That man really needed to share his story, and I thought I can just listen […] and that gives me the feeling that I am not just listening to, but really helping people, allowing them to blow off steam, and I think that is my role as a clinical clerk […] apparently I am allowed to take this time, but as doctor you are not. (Saar, Year 5)

Where in the beginning of medical training, Saar just tried to ‘get used to it a little’ and ‘to look past it’ (Saar, Year 1), she now had found a workaround to make emotions be part of her professional role, not necessarily by downplaying them, but by integrating them into her professional ‘face’. In close interaction with the clinical environment, Saar managed to develop a professional identity that is inclusive of emotion.

The final example we want to present here is the story of Suzanne, exemplifying those students who were sensitive to and felt comfortable with expressing emotions right from the beginning. They talked much with others, not necessarily to get help or better understand their experiences, but to preserve the right balance between involvement and self‐preservation. These students developed themselves while observing others, making deliberate choices for themselves about how to build on these observations. In her first clinical placement in the hospital, after having witnessed a major surgery, that ‘I felt was really impressive’, Suzanne explicitly stated that she needed to share her emotions with others:

It helps me to talk about it, because, when I do not share it with others, then it will stay inside me, and I think that this is natural, it is not really bottling up, it is not a kind of suppressing, but it stays with you; but when you talk about it, then it will more easily find a place, so for me it is important to talk about it. (Suzanne, Year 1)

She realised that she needed to learn how to balance involvement and detachment, saying:

I can get really involved, and I need to make sure that I do not take what happens here home with me. (Suzanne, Year 1)

A few years later, after having seen other people losing their empathy, she deliberately decided to do things differently from what she observed in practice:

I hope that I will preserve [my empathy], but I don't know if I will manage, but I think that, in my work, that I can keep it, if I allow myself to do other things I like to do outside work as well, that I am not only working, because when I see the people who are working here so much, I get the feeling that they are becoming numb sometimes, and I really don't want that to happen to me. (Suzanne, Year 4)

Just like Saar, Suzanne was also able to find a specific meaning in her role as a clerk, which she explained after having been able to comfort a patient during a very painful procedure:

Finally, that woman said that she was so glad that I had been with her, by your presence it might have been less painful, and I really like that, when people say this to you, I think, as a clerk, you sometimes can have a specific, social role. (Suzanne, Year 4)

By taking up this role, Suzanne was able to develop an attitude of involvement, identifying already as a health care professional but with a specific role, crafting an emotional and social space.

## Discussion

The socialisation of emotion in the process of becoming a doctor happens in a complex interplay between student and context. We identified two modes of emotional socialisation, explicit and implicit teaching about emotions, the latter including how the people observed by students express their emotions and how they respond to the emotions expressed by students. Although the main message conveyed to students still seems one about hiding or suppressing emotion, we found that students were able to identify and build upon the emotional expression and responses they observed in positive role models and managed to create their own opportunities to express their emotions. We also found that students responded differently to what they were taught, what they observed and how others responded to them. They differed in how they were influenced by or interacted with the social context. By presenting the stories of four exemplar students, we have illustrated this mutual interaction between student and context and how this unfolds over time.

The dominant conversation about the socialisation of emotion in medical school is one about power.^19^, ^20^, ^21^, ^22^ Our study adds to this conversation by showing how, within this field of power, students are able to craft an emotional space to express or act upon emotion. For some students, who feel uncertain or insecure, this might be fairly complicated, as we illustrated in the story of Lineke. Those students need a supportive environment, in which preceptors or supervisors offer actual support and opportunities to express their emotions. Even if they do not dare to ask this for themselves, we found that they were able to act upon their emotions when they felt responsible for patients. Both Lineke and Bernard, who did not readily express their emotions, were able to respond to their feelings when acting upon this responsibility. They referred to their specific responsibilities in the care of patients, for instance when observing injustice or unprofessional behaviours. Other students, as exemplified in the narratives of Saar and Suzanne, claimed a specific role for themselves as clinical clerks, being able to spend time really listening to the stories of patients and as such being of important added value to the health care system, although acknowledging that this might no longer be possible when they were doctors. These students actively created opportunities for expressing emotions as part of their personal and professional development. This may be one possible answer to the invitation of Dornan et al.^22^ to ‘explore how it can be made legitimate to talk about physicians’, residents’ and medical students’ emotions, and how those emotions link to power and identity development’.

How students expressed and responded to emotions indeed was closely related to their image of self, which parallels what we have shown before.^2^ In this longitudinal follow‐up of students about whom we have published before,^2^, ^15^ we found that the previously identified ‘paradigms of lived experience’ were still present. Lineke represents the ‘feeling insecure’ paradigm, but over the years, she managed to move beyond just feeling uncertain and take up responsibility for patients. Although Bernard, representing the complying paradigm, does not comply with the environment in all situations (actually he was able to stand up for a patient very explicitly) he seems to be complying with a rather fixed image of being a doctor and chooses to present himself as non‐emotional throughout the whole 6‐year period, indicating a certain detachment from emotional experiences. By doing so, he strongly aligns with the dominant discourse of competence.^21^ Saar and Suzanne, by contrast, representing the ‘developing’ and ‘participating’ paradigms, are actively involved in emotional exploration and able to ask for help or even to create emotional space for themselves and others. In doing so, they are much more receptive to discourses of caring^21^ or emotion.^22^ These four ‘paradigmatic’ students show how they all are socialised differently and how they are all able to craft their social environment in different ways. Thus, although students are both explicitly and implicitly taught to hide or suppress their emotions, which fits previous accounts of the role power plays in the development of students,^21^, ^22^ our study shows how students are not just passively being socialised but are able to find ways of expressing and acting upon emotion that fit their developing professional identities. The different ways of enacting this process over time again suggest that the different paradigms of lived experience are applicable throughout medical training, as was recently also suggested by other authors.^17^

### Strengths and limitations

This is one of the first longitudinal qualitative studies in which students were followed throughout the full 6‐year medical school period. Having one and the same interviewer (EH) can be considered a major strength, as this allowed for the development of rapport between researcher and participants. By contrast, the use of just one interviewer will always imply certain blind spots.^30^ For instance, as EH works as an elderly care physician, she may have been particularly interested in students’ experiences with complex situations in the care of older patients with chronic illness and many co‐morbidities, instead of focusing on acute in‐hospital situations that might be really exciting for trainees. To keep an open eye, she actively sought discussions not only with the participants themselves, but also with fellow researchers inside and outside our own research team. Having carried out all the interviews, she asked a medical student (MB) to take the lead in carrying out the analyses, actively seeking the medical student perspective. Despite the fact that we included a small sample from only one university, we think the results are transferable to other Dutch universities and, at least in part, also in an international context.

### Implications for medical education and future research

We see some important implications for medical education and future research. First, medical educators and clinical teachers need to realise that students differ in how they respond to and what they need from their environment. Students who feel uncertain and overwhelmed may initially need a high amount of explicit support to feel comfortable with being in clinical practice and sharing emotions. For ‘complying’ students, specific interventions may be warranted to help them become aware of and respond to emotions, if not their own, then at least those of patients and colleagues. Students from the developing and participating paradigms seem to be particularly accommodated by being allowed to spend time talking about emotions with both colleagues and patients. Future research might benefit from the longitudinal follow‐up of larger groups of students and may want to further explore different types of support or intervention.

Secondly, although students talked about hiding their emotions, we found two notable exceptions: (i) when students felt the need to stand up for patients, they were able to show their emotions and act upon them, and (ii) students were able to craft specific roles for themselves as clerks in providing emotional support to patients. This might offer another important implication for medical education. By giving students real and authentic responsibility for patients, even when this is only possible for small aspects of the whole care of a patient, they might be more likely to act upon their emotions in the best interests of patients. In this process, teachers and clinical supervisors need to provide support, attend to students’ emotional responses and offer opportunities to share emotions.^8^, ^22^

## Contributors

all authors directly participated in the writing and revision of the article. MB primarily analysed the data, drafted the first interpretations and wrote the first drafts of the paper. KD made substantial contributions to the interpretation of the data and critically revised the drafts; JdV contributed substantially to the interpretation of the data and critically revised the drafts. EH was responsible for the conception and design of the study and for collecting the data, and supervised the analysis and the writing process. All authors have approved the final version to be submitted.

## Funding

none.

## Conflicts of interest

none.

## Ethical approval

at the start of this longitudinal study, ethical approval for education research was not yet required in the Netherlands, and therefore this project was not taken under consideration by the institutional review board.
